# Advances in Cell Wall Dynamics and Gene Expression in Postharvest Fruit Softening

**DOI:** 10.3390/plants14182831

**Published:** 2025-09-10

**Authors:** Xumin Wang, Da Zhang, Tiantian Liu, Zhuo Yan, Xinmei Ji, Yusheng Li, Yaqin Wu, Hehe Cheng, Yingjie Wang, Jianchao Cui, Yongjie Wu, Long Chen

**Affiliations:** 1 Changli Institute of Pomology, Hebei Academy of Agriculture and Forestry Sciences, Qinhuangdao 066600, China; 15383822971@163.com (X.W.); d.zhang@nwafu.edu.cn (D.Z.); 13731396625@163.com (Z.Y.); jxm1008@163.com (X.J.); liyusheng1980@126.com (Y.L.); yaqin_wu@163.com (Y.W.); chenghehe2008@163.com (H.C.); 17733503931@163.com (Y.W.); cjc19880320@126.com (J.C.); 2 Xianyang Academy of Agriculture Sciences, Xianyang 712034, China; ltt786@163.com

**Keywords:** fruit quality, shelf life, polysaccharide metabolism, water loss, transcriptional regulation

## Abstract

Postharvest fruit softening is a critical determinant of fruit shelf life, significantly influencing mechanical damage susceptibility, pathogen invasion, and consumer preference. Collectively, these factors lead to substantial losses in the fruit industry. The structural modifications of cell wall and cuticle during ripening primarily govern fruit softening. The objective of this review is to synthesize recent advances and provide a comprehensive analysis of the molecular mechanisms underlying this process. In this review, we provide a comprehensive analysis of cell wall composition and softening-associated cell wall remodeling proteins. We examine recent advances in manipulating single or multiple genes encoding cell wall-modifying proteins that influence fruit softening, and identify key transcription factors regulating the expression of these gene networks. This review synthesizes current understanding of the molecular mechanisms governing fruit ripening, providing a foundation for future research in postharvest biology.

## 1. Introduction

The transition of fruits from commercial harvest maturity to edible ripeness is referred to as the postharvest ripening process, which simultaneously constitutes their storage period. Fleshy fruits exhibit considerable variation in structure, development, and biochemical makeup across different species. Nevertheless, their postharvest ripening processes are highly coordinated and share many common characteristics. During postharvest ripening, fruits undergo a series of physiological and biochemical changes, that enhance their appeal and nutritional value for seed-dispersing animals, and in the case of cultivated species, for human consumers [1]. Key changes include the buildup of sugars, pigments, and flavor or aromatic compounds, along with a reduction in firmness [2].

The postharvest softening of fruits represents one of the most significant alterations during fruit storage. On one hand, fruit firmness directly affects sensory texture; on the other hand, it serves as an important indicator of fruit quality and market value [3]. Fruit softening is due in large part to the controlled breakdown and modification of cell wall polysaccharides and water loss [2,4]. These postharvest mechanisms are controlled by evolutionarily conserved and convergent regulatory networks involving transcription factors and hormones [5]. In this article, we consider recent advances in cell wall modification and cuticle biology that address controlling fruit softening and extending storage and shelf life, providing insights into the molecular mechanisms underlying fruit postharvest ripening.

## 2. The Process of Fruit Softening

As living biological systems, postharvest fruits maintain essential metabolic activities, with respiration serving as the predominant physiological process that critically influences both fruit quality attributes and storage performance [6]. Based on distinct respiratory patterns, fleshy fruits are categorized into two types: climacteric fruits (e.g., apples, bananas, and kiwifruits) and non-climacteric fruits (e.g., grapes, strawberries, and cherries). Fruits may be crisp like apples or very soft like peaches and berries, but they all soften when ripening [2]. The textural changes associated with fruit softening reflect composite effects of primary cell wall and middle lamella modifications (impacting structural stability and cellular adhesion) and decreasing turgor within cells [7,8].

**Figure 1:**
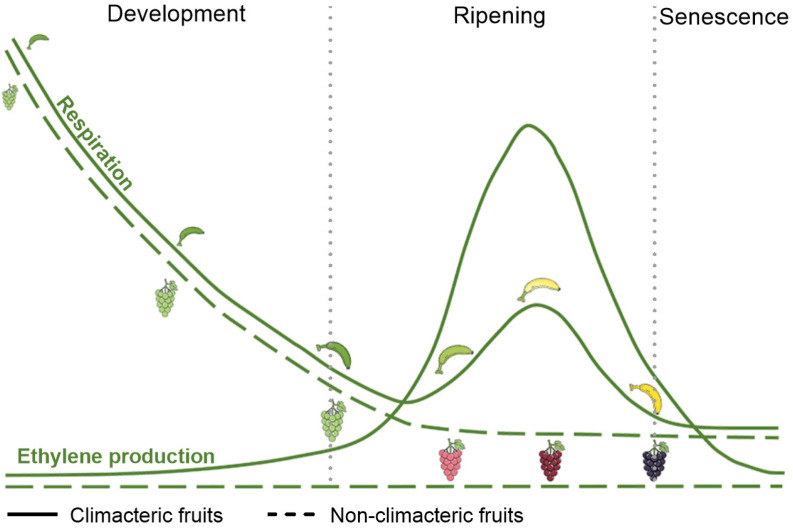
Shifts in respiratory rate and ethylene production in climacteric or non-climacteric fruit during development, maturation, and senescence [9]. Solid lines represent climacteric fruits (e.g., apples, bananas, and kiwifruits), and dashed lines correspond to non-climacteric fruits (e.g., grapes, strawberries, and cherries).

In climacteric fruits, ethylene concentration remains extremely low during developmental stages while respiratory rate progressively declines. Upon entering the ripening phase, ethylene production gradually increases to reach its peak level, concomitant with the occurrence of respiratory climacteric [9]. Following the respiratory climacteric, both ethylene production and respiratory rate decline, accompanied by significant deterioration in fruit quality and storability. The accelerated respiratory activity rapidly depletes nutritional reserves, leading to quality degradation and progressive loss of edibility [10,11]. In non-climacteric fruits, relatively low levels of endogenous ethylene still participate in regulating the postharvest ripening, despite the absence of a respiratory climacteric [12] (Figure 1).

## 3. Cell Wall and Fruits Softening

### 3.1. Composition of the Cell Wall

**Figure 2:**
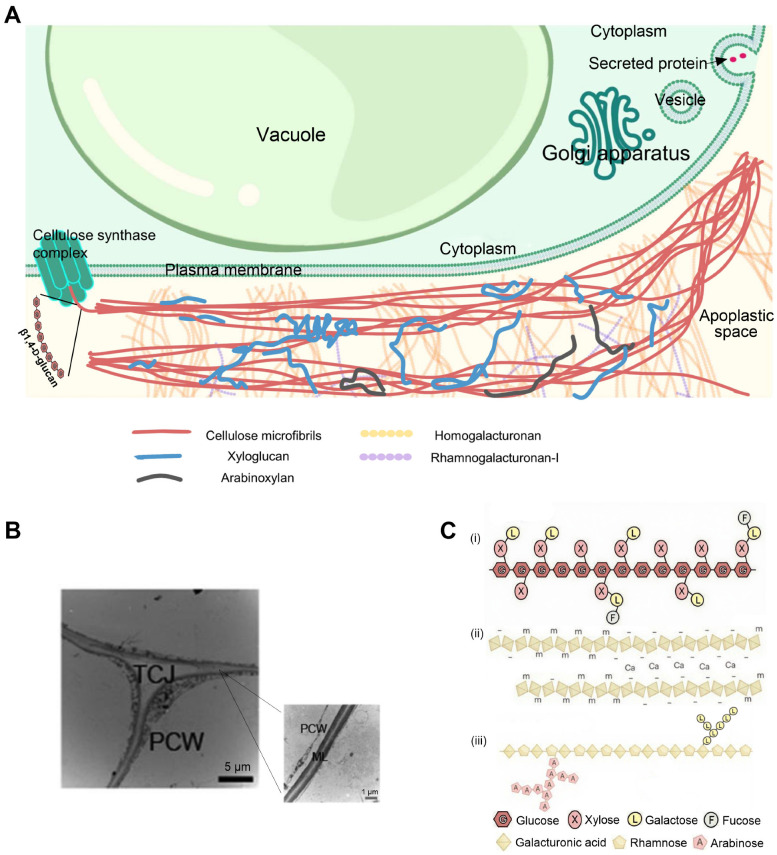
(**A**)**.** The model of the growing cell wall [14]. Cellulose microfibrils establish lateral connections, creating bundles and an integrated framework. Xyloglucans associate with cellulose surfaces in extended or folded conformations or are enclosed within cellulose aggregates. Pectins generate a gel-like matrix that encompasses cellulose and xyloglucan. (**B**). Transmission electron micrographs of TCJ (**left**) and ML (**right**) [33]. The ML between adjacent cells is rich in de-esterified homogalacturonan (HGA). PCW, primary cell wall. ML, middle lamella. TCJ, tricellular junctions. The bar (**left**) indicates 5 μm, and the bar (**right**) indicates 1 μm. (**C**). Schematic diagrams of molecular structures of several dominant cell wall polysaccharides [14]. (**i**) Xyloglucans. (**ii**) Homogalacturonan. (**iii**) Rhamnogalacturonan-I.

The plant cell wall is a rigid, multilayered structure that provides mechanical support and gives the cell its shape and form (Figure 2A) [13]. Plant cell wall consists of the primary cell wall, secondary cell wall, and middle lamella [14]. The middle lamella forms a sticky pectin layer outside the primary wall, acting as nature’s glue that bonds plant cells together [15]. After the primary wall stops expanding, some cells deposit a thick, rigid secondary wall inside the primary wall structure, a phenomenon most pronounced in woody tissues [16]. The softening of fruits is primarily related to the modifications of the primary wall and middle lamella, affecting wall strength and intercellular adhesion [2].

The primary wall contains strong and rigid cellulose microfibrils embedded in a hydrated matrix made of polysaccharides classified as pectins and hemicelluloses [17,18]. Cellulose is made of many parallel β1,4-D-glucan chains (Figure 2A). These chains are made by cellulose synthase complexes in the cell membrane, then form stiff microfibrils outside the membrane. The cellulose synthase enzyme has two main parts: a glucose-adding site inside the cell and a tube that moves the growing chain outside. It takes glucose from UDP-glucose inside the cell and adds it to the chain’s growing end [19].

Xyloglucan, the primary hemicellulose in primary walls (with minor arabinoxylan content), possesses a β1,4-linked glucan backbone (like cellulose) with xylose residues linked to three glucose residues per tetrameric repeat (Figure 2C). Xylose residues can be decorated with diverse sugar groups including galactose and fucose in varying patterns. Branched sugar groups create spatial hindrance that inhibits dense packing of xyloglucan polymers while maintaining their cellulose adhesion capacity [20].

Homogalacturonan (HGA), the predominant structural component of pectin, possesses a α1,4-linked D-galacturonic acid backbone (Figure 2C). During biosynthesis in Golgi apparatus, the majority of homogalacturonan residues undergo carboxyl group methylation [21]. The methyl esters can be partially eliminated by pectin methylesterase (PME), which makes HGA susceptible to depolymerization by endo-polygalacturonase (PG) and pectate lyase (PL) [22,23]. Processive de-esterification by PME also generates linear segments of six or more residues that sometimes form calcium-mediated ionic crosslinks with neighboring chains, thereby stiffening the pectic matrix [24,25]. Rhamnogalacturonan-I (RG-I), another important pectin component, consists of a linear chain with alternating rhamnose and galacturonic acid molecules (Figure 2C). The RG-I polymer often carries multiple oligosaccharide side chains and can form covalent bonds with both homogalacturonan and glycoproteins [26]. These structural features enable RG-I to participate in middle lamella adhesion and influence various cell wall characteristics [27].

Beyond the classical tethered network and pectic hydrogel models, a wall model was recently developed [14]. Cellulose microfibers join together through different physical links to form tight bundles and an interwoven network, where direct fiber-to-fiber junctions are strongest [28,29,30]. Xyloglucans adopt various conformations, and they bound to cellulose surfaces in extended or coiled forms or get caught inside cellulose bundles. They can also link multiple cellulose fibers like ropes [14]. Pectins assemble into a hydrogel matrix encapsulating cellulose and xyloglucan [31]. Xyloglucan and pectins may regulate wall mechanics by controlling cellulose’s assembly into a load-bearing nanostructure [30]. The matrix polysaccharides are flexible and have unique traits, molecular connections, dynamic changes, and structural functions, and the matrix also contains trace amounts of structurally diverse proteins and proteoglycans with diverse proposed functions [32].

### 3.2. Cell Wall Remodeling Proteins

Many cell wall remodeling proteins (CWRPs) are secreted into the apoplast to modify these wall polysaccharides during postharvest ripening. These include the hemicellulose-modifying expansin (EXP), endo-1,4-β-glucanase (EGase), and the xyloglucan endo-transglycosylase/hydrolase (XTH), and the pectin-modifying enzymes PL, PG, PME, rhamnogalacturonan lyase (RGL), β-galactosidase (BGAL), and α-arabinofuranosidase (AFase) [34]. The corresponding HGA and xyloglucan breakdown, HGA demethylesterification, and RG-I side-chain removal reduce intercellular adhesion [2]. The influence of these enzymatic activities on wall properties is determined by their temporal expression patterns, quantitative activity levels, and the species-specific wall composition. The composition of the cell wall is species dependent. The structural and compositional diversity of microalgal cell walls has been systematically cataloged [35]. *Poaceae* species exhibit a distinctive capacity to synthesize (1,3;1,4)-β-glucans with mid-range ratios of cellotriosyl to cellotetraosyl units, which may explain the widespread occurrence of these polysaccharides across the family [36]. Variations in cell wall architectural properties among different switchgrass (*Panicum virgatum*) genotypes directly affect their accessibility to hydrolytic enzymes [37]. Ripening-related *PG1* was specifically detected in fast-softening ‘Royal Gala’ but absent in ‘Scifresh’, confirming its cultivar-dependent expression and pivotal role in apple texture determination [38,39]. Goulao et al. systematically profiled the enzymatic activity patterns of cell wall-modifying enzymes throughout both growth and ripening phases. Their comprehensive analysis revealed that while distinct enzyme isoforms may mediate specific developmental processes, the overall coordination of fruit growth and ripening appears to be governed by members of the same families of cell wall-modifying enzymes [40].

### 3.3. Modulators Involved in the CWRPs-Mediated Regulatory Network of Fruit Softening

Efforts to directly modify genes encoding homogalacturonan-depolymerizing enzymes have proven effective in fruit softening regulation. The slow-softening ‘Scifresh’ variety exhibited diminished endo-polygalacturonase and pectin methylesterase activity in early development phases, correlating with enhanced cellular cohesion and delayed softening kinetics [41]. Manipulation of these individual genes triggers cascading effects on fruit ripening and softening [23]. *MdPG1*, an endo-polygalacturonase gene, functions as a key marker for fruit softening in apples. While tomato ripening-related *SlPG2a* is induced exclusively by very low ethylene concentrations, apple *MdPG1* expression responds not only to ethylene but also to extended cold temperatures during postharvest storage in the absence of ethylene [42,43]. Moreover, *MdPG1* expression remained minimal in firmness-retaining cultivars following extended cold storage, regardless of ethylene synthesis [44]. In short-shelf-life ‘Golden Delicious’, *MdPG1* expression in the cortex increased rapidly during ripening [45]. Up-regulation of *MdPG1* enhances pectin hydrolysis in the middle lamella, elevating water-soluble pectin levels and accelerating postharvest softening in apples [23]. Down-regulation of *MdPG1* reduced pectin solubilization, pectin depolymerization, and fruit softening [46]. MdEIL2 and MdCBF2 directly control the expression of *MdPG1*, working together to regulate ethylene-induced apple fruit softening during cold storage [43]. MADS-box transcription factors also play important roles in fruit softening regulation. MdMADS6, MdMADS8, and MdMADS9 directly regulate *MdPG1* transcription in apples [47]. Ethylene-induced MdPUB24 mediates the ubiquitination and subsequent degradation of MdNAC72. Moreover, ethylene stimulates MdMAPK3 to phosphorylate MdNAC72, further diminishing its inhibitory effect on *MdPG1* expression. These coordinated mechanisms ultimately accelerate fruit softening in postharvest storage [3]. The MdZFP3 assembles with co-repressor MdTPL4 and chromatin modifier MdHDA19 to form a repression complex that epigenetically silences cell wall degradation genes (*MdPG1*, *MdPL5*, *Mdβ-Gal9*, *Mdα-AFase2*, *MdXET1*, and *MdEXP8*). Additionally, E3 ubiquitin ligase MdEAEL1 targets MdZFP3 for ubiquitin-mediated degradation, resulting in disassembly of the repression complex [5]. The 8 bp deletion in the *MdERF3* promoter abolished MdDOF5.3 binding, leading to down-regulation of *MdERF3*. This loss of repression enhanced the expression of *MdPGLR3*, *MdPME2*, and *MdACO4*, ultimately impairing fruit firmness and crispness retention. Similarly, a 3 bp deletion in the *MdERF118* promoter attenuated MdRAVL1 binding, suppressing *MdERF118* expression. Consequently, *MdPGLR3* and *MdACO4* were up-regulated, further contributing to fruit softening [48]. MdPL5 promoted fruit softening in apples and tomatoes. MdNAC1-L acted as a transcriptional activator that positively regulates fruit ripening and softening through promoting the expression of cell wall degradation genes *MdPL5* and *MdPG1*, and ethylene biosynthesis genes *MdACS1* and *MdACO1* [49].

HGA depolymerization serves as a key driver of fruit softening in both crisp and soft fruit, though the relative contributions of PG- versus PL-mediated degradation pathways vary significantly across species [50]. In strawberry, silencing either the PL gene *FaPLC* or the PG genes *FaPG1* and *FaPG2* significantly inhibited fruit softening [51,52,53]. Overexpression of grapevine *VvPL11* in tomato significantly elevated ethylene production and reduced fruit firmness, accompanied by decreased propectin content and increased water-soluble pectin accumulation [54]. VIGS-mediated silencing of *PpPG21* and *PpPG22* in peach fruit substantially decreased PG activity and preserved firmness during postharvest storage [55]. Down-regulation of *SlPL* expression increased cellulose and hemicellulose content while decreasing water-soluble pectin, leading to enhanced intercellular adhesion, substantial reduction in fruit softening, and improved resistance to biotic stress in tomato [56]. PpERF/ABR1 up-regulates *PpPG* expression to promote peach fruit softening [57]. The ethylene-down-regulated transcription factor SlERF.F12 associates with SlTPL2 via its C-terminal EAR motif and recruits histone deacetylases SlHDA1/3 into a tripartite complex. This complex reduces histone acetylation at the promoter regions of cell wall disassembly-related genes including *SlPG2a* and *SlPL*, thereby repressing their expression and consequently delaying fruit softening [58].

The cell wall loosening and swelling induced by EXP may promote further wall modification by increasing substrate accessibility for other enzymes, including PG and PL [2]. Tomato *SlPG2a* affects pectin metabolism but contributes little to softening when inhibited [59]. Co-suppression of both *SlPG2a* and *SlEXP1* results in substantially increased tomato fruit firmness relative to individual suppression of either gene [60]. Suppression of *SlEXP1* in tomato significantly elevated fruits firmness throughout ripening. This suppression markedly inhibited late-stage polyuronide depolymerization while having minimal effect on the degradation of hemicelluloses [61]. Apple *MdEXLB1*-overexpressing transgenic tomato lines exhibited multiple phenotypic alterations, including reduced plant height, accelerated reproductive development, and advanced fruit maturation, and overall faster ripening progression [62]. *MdEXP7* is also linked with fruit softening [63]. In banana (*Musa acuminata*), the zinc finger transcription factors MaC2H2-1 and MaC2H2-2 up-regulate the expression of cell wall-modifying genes, including *MaEXP-A2*, *MaEXP-A8*, and *MaSUR14*, thereby accelerating postharvest ripening and softening [64]. SlLOB1 activates *SlEXP1* and *SlPL* expression, and its repression delays fruit softening and extends shelf life [65]. Silencing of *MdBBX25* accelerated fruit softening in apple by increasing ethylene synthesis and elevating the expression of cell wall-related genes, including *MdPG*, *MdCEL*, and *MdEXPA8* [66].

The XTH plays crucial roles throughout fruit development. XTH activity peaked in both apple and kiwifruit fruits within two weeks of post-anthesis, then declined sharply before rebounding during fruit expansion. In apples, activity peaked at harvest then declined postharvest, while kiwifruit core tissue showed similar patterns but continued increasing until ripening. Outer pericarp XTH increased only during postharvest ripening [67]. Persimmon *DkXTH8* overexpression induces cellular fragility, and enhances membrane leakage and oxidative damage, while promoting premature leaf aging and fruit texture softening in transgenic plants [68]. In strawberry, transient overexpression of *FvXTH9* or *FvXTH6* promoted ripening and softening [69]. The knock-out of SlXTH5 in tomato resulted in slightly firmer fruit pericarp [70]. Overexpression of *MdXTHB* in ‘Golden Delicious’ and ‘Fuji’ apples resulted in accelerated softening and an earlier ethylene production peak [71]. Overexpression of *MdXTH2* and *MdXTH10* in tomato fruits increased the expression of ethylene-related genes (*ACS2* and *ACO1*) and cell wall-modifying enzymes (*XTHs*, *PG2A*, *Cel2*, and *TBG4*) [72]. Ethylene-repressed MdWRKY31 interacts with ethylene-induced MdNAC7, relieving MdWRKY31-mediated transcriptional repression of *MdXTH2*. This molecular interplay promotes fruit softening through loosening the cell wall and facilitating catabolic activities within the cellulose–xyloglucan matrix [73].

Fruit cell wall galactosyl levels show a correlation with tissue firmness [74]. Transient silencing of *PpBGAL10* or *PpBGAL16* in peach inhibited ethylene production and subsequent *PpPG* expression, leading to delayed fruit ripening and softening [75]. Overexpression of β-galactosidase *DkGAL1* induced earlier ethylene evolution, which subsequently promoted pigmentation shift and textural softening [76]. Silencing *β-galactosidase 4* reduces fruit softening in tomato [77]. Suppression of FaβGal4 elevates cell wall galactose content and decreases fruit softening in strawberry [78]. Ethylene-induced MdAP2-like promotes fruit softening by activating *Mdβ-GAL18* expression, which increases β-galactosidase activity and free galactose levels in apple fruits [79]. Ethylene-induced MdZF-HD11 also positively regulates the expression of *Mdβ-GAL18* to promote the postharvest softening in apple [7]. α-AFase catalytic activity also shows a persistent positive association with fruit hardness, which grew with a lengthened storage time. *MdAF3* expression is increased in fruits harvested from plants persistently exhibiting mealiness characteristics and elevated α-AFase activity [80]. MdDof43 activates *Mdβ-Gal2* and *Mdα-AF3* transcription, enhancing cell wall degradation and accelerating fruit softening [81], and the αAFase, β-Gal, and PG can complement each other in pectin degradation, thereby reducing cell adhesion and promoting fruit softening [82]. The key factor in apple anthocyanin regulation, MdbHLH3, also enhances the αAFase, β-Gal, and PG activities during postharvest storage [82].

Hormones play a crucial role in regulating fruit softening. In addition to ethylene as mentioned above, abscisic acid (ABA) and gibberellins (GAs) are also involved in this process. Abscisic acid accelerates postharvest softening of blueberry fruit by enhancing cell wall metabolism [83]. Gibberellins are involved in fruit ripening and softening by mediating multiple hormonal signals [84]. Interestingly, a newly identified macromolecule, class 1 non-symbiotic hemoglobin (MdHb1), promoted fruit softening through mediating the depolymerization of protopectin to water-soluble pectin. Two metabolites, D-galacturonic acid and D-glucuronic acid, suppress the transcription of *MdHb1* through the MdMYB2/MdNAC14/MdNTL9-MdHb1 regulatory module, thereby delaying fruit softening in apple [85].

Certainly, the role of hormones in regulating the ripening/softening of climacteric and non-climacteric fruits exhibits significant differences. In climacteric fruits, ethylene and ABA interact to coordinately regulate these processes, whereas in non-climacteric fruits, ripening/softening are predominantly controlled by ABA in an ethylene-independent manner, as extensively reviewed by Kou et al., making the investigation of these regulatory differences a highly promising research direction [86].

## 4. Water Loss

Postharvest water loss from the entire fruit considerably accelerates softening [87]. Mature fruit generally lack functional stomata, which turn into lenticels covered by periderm tissue [23,88]. Composed of a matrix of the polyester cutin impregnated with various waxes or phenolics, the fruit cuticle serves as nature’s waterproof coating that minimizes dehydration [89]. A reduction in cellular turgor pressure, resulting from solute and water movements between the apoplast and symplast, further contributes to fruit softening [2,90].

Epidermal wax disruption treatments significantly increased postharvest weight loss in peach, plum, and citrus [91,92,93]. Moreover, blueberries subjected to epidermal wax disruption treatments exhibited rapid cell wall degradation postharvest, leading to a significant decline in fruit firmness. In contrast, berries with intact natural wax coatings maintained extended shelf life [94]. The thicker cuticle and higher wax content in citrus fruits also reduce pathogen invasion and decrease fruit maceration [95].

Postharvest preservation treatments can alter epidermal wax composition, thereby influencing fruit storability. A 1-MCP treatment significantly inhibited the decline of cuticular wax content in apple fruit, while concurrently delaying the reduction in fruit firmness and loss of nutritional compounds [96,97]. Ethylene treatment induced increased cuticular wax accumulation in citrus fruit, concomitant with structural modifications of wax morphology and reduced incidence of brown spot disease [98]. The distinctive morphological structures and biological functions of cuticular wax play critical roles in the application of fruits postharvest preservation technologies. Various wax coating applications enhance marketability and extend storage duration in commercial fruits such as citrus and apples [89,98,99].

A correlation was observed between alterations in the cell wall architecture and dehydration processes during fruit softening. Peel cell walls of *MdPG1*-overexpressing fruits exhibited thinner with reduced cell wall content, along with increased water-soluble pectin. The outer cell layers became disorganized, with cells separating widely and large air spaces below the epidermis, resulting in increased water loss in apples [23,46]. Overexpression of *MdPG1* also reduces fruit skin fracture force, leading to cuticular microcracking and ultimately full-scale fruit cracking [23,100]. Down-regulation of *SlPL* expression in tomato induced tighter cellular packing and decreased transpirational water loss [56]. Moreover, specific genetic loci regulate fruit water loss without significantly altering cuticular architecture. Following harvest, *SlLOB1*-silenced fruit exhibited diminished tissue collapse and markedly decreased water loss, while cuticle thickness remained unchanged [65]. Overexpression of *SlFSR* significantly reduced the shelf life and increased water loss in stored fruits by promoting the expression of cell wall genes, including *SlPG*, *SlPL*, and *SlEXP1* [101]. Increased skin cracking may result from altered cell wall structure, caused by β-galactosidase *TBG6* silencing, increased *SlEXP1*/*SlPG2a* expression, or heterologous *VvPL1* pectate lyase overexpression in tomato, which impair the cuticle’s barrier function, allowing water vapor to penetrate the skin [102,103,104,105]. Overexpression of the *myo*-inositol monophosphate gene *SlIMP3* enhanced wall thickening and decreased fruit softening, as well as water loss [106]. To facilitate the interpretation of the review data, we have summarized the main genes/enzymes involved in fruit softening in Supplementary Table S1.

## 5. Conclusions and Prospects

**Figure 3:**
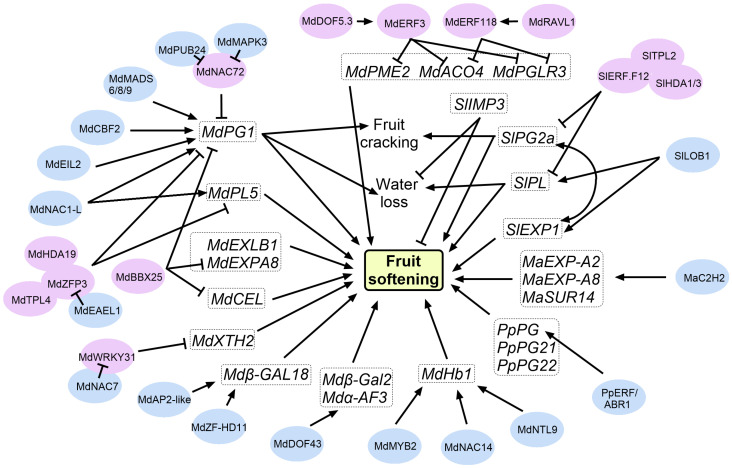
A model for the cell wall remodeling protein-mediated regulatory network of fruit softening. Arrows and T bars indicate promoting and inhibitory effects, respectively. Blue and pink colors represent positive and negative regulators of fruit softening, respectively.

Pectin depolymerization, primarily facilitated by PG or PL, is a well-established key driver of fruit softening in many species (Figure 3, Supplementary Table S1). Significant progress has been made in understanding that the loss of primary cell wall integrity, intercellular adhesion, turgor pressure, and water content collectively shape the final fruit texture. This knowledge has been beneficially applied to distinguish the phenotypic outcomes of softening: for instance, firm fruits retain robust cell walls and high turgor, while weakened adhesion promotes mealiness. Beyond canonical enzymes, the exploration of various other cell wall-modifying proteins has expanded our understanding of texture regulation, although their mechanisms remain an active area of research.

Building on this foundation, recent studies have begun to characterize the roles of expansion (EXPs) and xyloglucan endotransglucosylase/hydrolases (XTHs) in fruit softening, moving beyond their well-defined functions in seed germination or herbaceous species [107,108]. These investigations represent a promising shift towards elucidating how diverse enzymatic activities coordinate in the complex process of cell wall remodeling (Figure 3, Supplementary Table S1). Furthermore, the recognition that softening is influenced by environmental signals (temperature, humidity, and light) has opened a new frontier: a major forthcoming challenge and opportunity lies in deciphering the molecular links between these environmental signals and the cell wall-modifying pathways [109].

The development of CRISPR/Cas9 technology has been a transformative change, enabling precise multiplexed gene editing to improve fruit quality traits—such as simultaneously enhancing water retention and reducing cell wall disassembly. This strategy offers a superior alternative to RNAi silencing, as it can generate a spectrum of natural partial-loss-of-function mutations without unintended side effects, presenting significant potential for biotechnological cultivar improvement. Concurrently, traditional breeding enhanced by GWAS and QTL mapping continues to be a pivotal and successful approach for quality improvement and gene discovery. Recently, we developed a new cultivar ‘Yuguan’ with high wax content and prolonged shelf life [110], which provides an excellent model for future research. While CRISPR/Cas9 is a powerful tool for fruit quality improvement, the elimination of transgenes remains a significant challenge in vegetatively propagated crops. Alternatively, manipulating upstream master transcription factors that regulate gene networks offers a more straightforward and commercially viable strategy. Looking forward, strategic perspectives for both research and application should prioritize these two avenues. These data-driven perspectives, grounded in contemporary technological advances, chart a clear course for developing the next generation of fruit cultivars with optimized postharvest quality.

## Supplementary Materials

The following supporting information can be downloaded at https://www.mdpi.com/article/10.3390/plants14182831/s1; Table S1. Modulators Involved in the CWRPs-Mediated Regulatory Network of Fruit Softening; Table S2. List of Abbreviations.

## References

[1] Migicovsky Z., Yeats T.H., Watts S., Song J., Forney C.F., Burgher-MacLellan K., Somers D.J., Gong Y., Zhang Z., Vrebalov J. (2021). Apple ripening is controlled by a NAC transcription factor. Front. Genet..

[2] Brummell D.A., Bowen J.K., Gapper N.E. (2022). Biotechnological approaches for controlling postharvest fruit softening. Curr. Opin. Biotechnol..

[3] Wei Y., Liu Z., Lv T., Xu Y., Wei Y., Liu W., Liu L., Wang A., Li T. (2023). Ethylene enhances MdMAPK3-mediated phosphorylation of MdNAC72 to promote apple fruit softening. Plant Cell.

[4] Wang D., Yeats T.H., Uluisik S., Rose J.K.C., Seymour G.B. (2018). Fruit Softening: Revisiting the Role of Pectin. Trends Plant Sci..

[5] Li T., Liu L., Yang G., Cai Y., Wang Y., Sun B., Sun L., Liu W., Wang A. (2025). Ethylene-Activated E3 Ubiquitin Ligase MdEAEL1 Promotes Apple Fruit Softening by Facilitating the Dissociation of Transcriptional Repressor Complexes. Adv. Sci..

[6] Win N.M., Yoo J., Kwon S.-I., Watkins C.B., Kang I.-K. (2019). Characterization of fruit quality attributes and cell wall metabolism in 1-methylcyclopropene (1-MCP)-treated ‘Summer King’ and ‘Green Ball’ apples during cold storage. Front. Plant Sci..

[7] Wang M., Wu Y., Zhan W., Wang H., Chen M., Li T., Bai T., Jiao J., Song C., Song S. (2024). The apple transcription factor MdZF-HD11 regulates fruit softening by promoting Mdβ-GAL18 expression. J. Exp. Bot..

[8] Johnston J.W., Hewett E.W., Hertog M.L. (2002). Postharvest softening of apple (*Malus domestica*) fruit: A review. N. Z. J. Crop Hortic. Sci..

[9] Ji Y., Xu M., Wang A. (2021). Recent advances in the regulation of climacteric fruit ripening: Hormone, transcription factor and epigenetic modifications. Front. Agric. Sci. Eng..

[10] Kevany B.M., Tieman D.M., Taylor M.G., Cin V.D., Klee H.J. (2007). Ethylene receptor degradation controls the timing of ripening in tomato fruit. Plant J..

[11] Alexander L., Grierson D. (2002). Ethylene biosynthesis and action in tomato: A model for climacteric fruit ripening. J. Exp. Bot..

[12] Zhu P., Xu L., Zhang C., Toyoda H., Gan S.-S. (2012). Ethylene produced by Botrytis cinerea can affect early fungal development and can be used as a marker for infection during storage of grapes. Postharvest Biol. Technol..

[13] Cosgrove D.J. (2016). Catalysts of plant cell wall loosening. F1000Research.

[14] Cosgrove D.J. (2024). Structure and growth of plant cell walls. Nat. Rev. Mol. Cell Biol..

[15] Daher F.B., Braybrook S.A. (2015). How to let go: Pectin and plant cell adhesion. Front. Plant Sci..

[16] Li W., Lin Y.-C.J., Chen Y.-L., Zhou C., Li S., De Ridder N., Oliveira D.M., Zhang L., Zhang B., Wang J.P. (2024). Woody plant cell walls: Fundamentals and utilization. Mol. Plant.

[17] Atmodjo M.A., Hao Z., Mohnen D. (2013). Evolving views of pectin biosynthesis. Annu. Rev. Plant Biol..

[18] Scheller H.V., Ulvskov P. (2010). Hemicelluloses. Annu. Rev. Plant Biol..

[19] Pedersen G.B., Blaschek L., Frandsen K.E., Noack L.C., Persson S. (2023). Cellulose synthesis in land plants. Mol. Plant.

[20] Zhang B., Gao Y., Zhang L., Zhou Y. (2021). The plant cell wall: Biosynthesis, construction, and functions. J. Integr. Plant Biol..

[21] Temple H., Phyo P., Yang W., Lyczakowski J.J., Echevarría-Poza A., Yakunin I., Parra-Rojas J.P., Terrett O.M., Saez-Aguayo S., Dupree R. (2022). Golgi-localized putative S-adenosyl methionine transporters required for plant cell wall polysaccharide methylation. Nat. Plants.

[22] Shi D., Ren A., Tang X., Qi G., Xu Z., Chai G., Hu R., Zhou G., Kong Y. (2018). MYB52 negatively regulates pectin demethylesterification in seed coat mucilage. Plant Physiol..

[23] Gunaseelan K., Schröder R., Rebstock R., Ninan A.S., Deng C., Khanal B.P., Favre L., Tomes S., Dragulescu M.A., O’Donoghue E.M. (2024). Constitutive expression of apple endo-POLYGALACTURONASE1 in fruit induces early maturation, alters skin structure and accelerates softening. Plant J..

[24] John J., Ray D., Aswal V.K., Deshpande A.P., Varughese S. (2022). Pectin self-assembly and its disruption by water: Insights into plant cell wall mechanics. Phys. Chem. Chem. Phys..

[25] Willats W.G., Orfila C., Limberg G., Buchholt H.C., van Alebeek G.-J.W., Voragen A.G., Marcus S.E., Christensen T.M., Mikkelsen J.D., Murray B.S. (2001). Modulation of the degree and pattern of methyl-esterification of pectic homogalacturonan in plant cell walls: Implications for pectin methyl esterase action, matrix properties, and cell adhesion. J. Biol. Chem..

[26] Tan L., Zhang L., Black I., Glushka J., Urbanowicz B., Heiss C., Azadi P. (2023). Most of the rhamnogalacturonan-I from cultured Arabidopsis cell walls is covalently linked to arabinogalactan-protein. Carbohydr. Polym..

[27] Saez-Aguayo S., Largo-Gosens A. (2022). Rhamnogalacturonan-I forms mucilage: Behind its simplicity, a cutting-edge organization. J. Exp. Bot..

[28] Park Y.B., Cosgrove D.J. (2012). A revised architecture of primary cell walls based on biomechanical changes induced by substrate-specific endoglucanases. Plant Physiol..

[29] Jarvis M.C. (2024). Forces on and in the cell walls of living plants. Plant Physiol..

[30] Cosgrove D.J. (2022). Building an extensible cell wall. Plant Physiol..

[31] Zhang Y., Yu J., Wang X., Durachko D.M., Zhang S., Cosgrove D.J. (2021). Molecular insights into the complex mechanics of plant epidermal cell walls. Science.

[32] San Clemente H., Kolkas H., Canut H., Jamet E. (2022). Plant cell wall proteomes: The core of conserved protein families and the case of non-canonical proteins. Int. J. Mol. Sci..

[33] Su G., Lin Y., Wang C., Lu J., Liu Z., He Z., Shu X., Chen W., Wu R., Li B. (2023). Expansin SlExp1 and endoglucanase SlCel2 synergistically promote fruit softening and cell wall disassembly in tomato. Plant Cell.

[34] Steinbrecher T., Leubner-Metzger G. (2018). Tissue and cellular mechanics of seeds. Curr. Opin. Genet. Dev..

[35] Alhattab M., Kermanshahi-Pour A., Brooks M.S.-L. (2019). Microalgae disruption techniques for product recovery: Influence of cell wall composition. J. Appl. Phycol..

[36] Burton R.A., Gidley M.J., Fincher G.B. (2010). Heterogeneity in the chemistry, structure and function of plant cell walls. Nat. Chem. Biol..

[37] Sarath G., Akin D.E., Mitchell R.B., Vogel K.P. (2008). Cell-wall composition and accessibility to hydrolytic enzymes is differentially altered in divergently bred switchgrass (*Panicum virgatum* L.) genotypes. Appl. Biochem. Biotechnol..

[38] Wei J., Ma F., Shi S., Qi X., Zhu X., Yuan J. (2010). Changes and postharvest regulation of activity and gene expression of enzymes related to cell wall degradation in ripening apple fruit. Postharvest Biol. Technol..

[39] Longhi S., Hamblin M.T., Trainotti L., Peace C.P., Velasco R., Costa F. (2013). A candidate gene based approach validates Md-PG1 as the main responsible for a QTL impacting fruit texture in apple (*Malus* × *domestica* Borkh). BMC Plant Biol..

[40] Goulao L.F., Santos J., de Sousa I., Oliveira C.M. (2007). Patterns of enzymatic activity of cell wall-modifying enzymes during growth and ripening of apples. Postharvest Biol. Technol..

[41] Ng J.K.T., Schröder R., Sutherland P.W., Hallett I.C., Hall M.I., Prakash R., Smith B.G., Melton L.D., Johnston J.W. (2013). Cell wall structures leading to cultivar differences in softening rates develop early during apple (*Malus* × *domestica*) fruit growth. BMC Plant Biol..

[42] Sitrit Y., Bennett A.B. (1998). Regulation of tomato fruit polygalacturonase mRNA accumulation by ethylene: A re-examination. Plant Physiol..

[43] Tacken E., Ireland H., Gunaseelan K., Karunairetnam S., Wang D., Schultz K., Bowen J., Atkinson R.G., Johnston J.W., Putterill J. (2010). The role of ethylene and cold temperature in the regulation of the apple POLYGALACTURONASE1 gene and fruit softening. Plant Physiol..

[44] Wakasa Y., Kudo H., Ishikawa R., Akada S., Senda M., Niizeki M., Harada T. (2006). Low expression of an endopolygalacturonase gene in apple fruit with long-term storage potential. Postharvest Biol. Technol..

[45] Li J., Zhu H., Yuan R. (2010). Profiling the expression of genes related to ethylene biosynthesis, ethylene perception, and cell wall degradation during fruit abscission and fruit ripening in apple. J. Am. Soc. Hortic. Sci..

[46] Atkinson R.G., Sutherland P.W., Johnston S.L., Gunaseelan K., Hallett I.C., Mitra D., Brummell D.A., Schröder R., Johnston J.W., Schaffer R.J. (2012). Down-regulation of POLYGALACTURONASE1 alters firmness, tensile strength and water loss in apple (*Malus* × *domestica*) fruit. BMC Plant Biol..

[47] Ireland H.S., Yao J.L., Tomes S., Sutherland P.W., Nieuwenhuizen N., Gunaseelan K., Winz R.A., David K.M., Schaffer R.J. (2013). Apple SEPALLATA1/2-like genes control fruit flesh development and ripening. Plant J..

[48] Wu B., Shen F., Wang X., Zheng W.Y., Xiao C., Deng Y., Wang T., Yu Huang Z., Zhou Q., Wang Y. (2021). Role of MdERF3 and MdERF118 natural variations in apple flesh firmness/crispness retainability and development of QTL-based genomics-assisted prediction. Plant Biotechnol. J..

[49] Su Q., Yang H., Li X., Zhong Y., Feng Y., Li H., Tahir M.M., Zhao Z. (2024). Upregulation of PECTATE LYASE5 by a NAC transcription factor promotes fruit softening in apple. Plant Physiol..

[50] Brummell D.A. (2020). Sensing when the wall comes tumbling down. J. Exp. Bot..

[51] Paniagua C., Ric-Varas P., García-Gago J.A., López-Casado G., Blanco-Portales R., Muñoz-Blanco J., Schückel J., Knox J.P., Matas A.J., Quesada M.A. (2020). Elucidating the role of polygalacturonase genes in strawberry fruit softening. J. Exp. Bot..

[52] Quesada M.A., Blanco-Portales R., Posé S., García-Gago J.A., Jiménez-Bermudez S., Munoz-Serrano A., Caballero J.L., Pliego-Alfaro F., Mercado J.A., Munoz-Blanco J. (2009). Antisense down-regulation of the FaPG1 gene reveals an unexpected central role for polygalacturonase in strawberry fruit softening. Plant Physiol..

[53] Jiménez-Bermudez S., Redondo-Nevado J., Munoz-Blanco J., Caballero J.L., López-Aranda J.M., Valpuesta V., Pliego-Alfaro F., Quesada M.A., Mercado J.A. (2002). Manipulation of strawberry fruit softening by antisense expression of a pectate lyase gene. Plant Physiol..

[54] Li W., He C., Wei H., Qian J., Xie J., Li Z., Zheng X., Tan B., Li J., Cheng J. (2023). VvPL11 is a key member of the pectin lyase gene family involved in grape softening. Horticulturae.

[55] Qian M., Xu Z., Zhang Z., Li Q., Yan X., Liu H., Han M., Li F., Zheng J., Zhang D. (2021). The downregulation of PpPG21 and PpPG22 influences peach fruit texture and softening. Planta.

[56] Yang L., Huang W., Xiong F., Xian Z., Su D., Ren M., Li Z. (2017). Silencing of Sl PL, which encodes a pectate lyase in tomato, confers enhanced fruit firmness, prolonged shelf-life and reduced susceptibility to grey mould. Plant Biotechnol. J..

[57] Cheng C., Liu J., Wang X., Wang Y., Yuan Y., Yang S. (2022). PpERF/ABR1 functions as an activator to regulate PpPG expression resulting in fruit softening during storage in peach (*Prunus persica*). Postharvest Biol. Technol..

[58] Deng H., Chen Y., Liu Z., Liu Z., Shu P., Wang R., Hao Y., Su D., Pirrello J., Liu Y. (2022). SlERF. F12 modulates the transition to ripening in tomato fruit by recruiting the co-repressor TOPLESS and histone deacetylases to repress key ripening genes. Plant Cell.

[59] Wang D., Samsulrizal N.H., Yan C., Allcock N.S., Craigon J., Blanco-Ulate B., Ortega-Salazar I., Marcus S.E., Bagheri H.M., Perez Fons L. (2019). Characterization of CRISPR mutants targeting genes modulating pectin degradation in ripening tomato. Plant Physiol..

[60] Shi Y., Li B.-J., Grierson D., Chen K.-S. (2023). Insights into cell wall changes during fruit softening from transgenic and naturally occurring mutants. Plant Physiol..

[61] Brummell D.A., Harpster M.H., Civello P.M., Palys J.M., Bennett A.B., Dunsmuir P. (1999). Modification of expansin protein abundance in tomato fruit alters softening and cell wall polymer metabolism during ripening. Plant Cell.

[62] Chen Y.-H., Bin X., An X.-H., Zhao D.-Y., Cheng C.-G., Li E.-M., Zhou J.-T., Kang G.-D., Zhang Y.-Z. (2022). Overexpression of the apple expansin-like gene MdEXLB1 accelerates the softening of fruit texture in tomato. J. Integr. Agric..

[63] Costa F., Van de Weg W., Stella S., Dondini L., Pratesi D., Musacchi S., Sansavini S. (2008). Map position and functional allelic diversity of Md-Exp7, a new putative expansin gene associated with fruit softening in apple (*Malus* × *domestica* Borkh.) and pear (*Pyrus communis*). Tree Genet. Genomes.

[64] Lai X., Zhu X., Chen H., Pang X., Chen W., Li X., Song Z. (2022). The MaC2H2-like zinc finger protein is involved in ripening and ripening disorders caused by chilling stress via the regulation of softening-related genes in ‘Fenjiao’ banana. Postharvest Biol. Technol..

[65] Shi Y., Vrebalov J., Zheng H., Xu Y., Yin X., Liu W., Liu Z., Sorensen I., Su G., Ma Q. (2021). A tomato LATERAL ORGAN BOUNDARIES transcription factor, SlLOB1, predominantly regulates cell wall and softening components of ripening. Proc. Natl. Acad. Sci. USA.

[66] Zhang K., Zhang D., Wang X., Xu X., Yu W., Wang C., Yuan Y., Yang S., Cheng C. (2024). Integrated analysis of postharvest storage characteristics of seven apple cultivars and transcriptome data identifies MdBBX25 as a negative regulator of fruit softening during storage in apples. Postharvest Biol. Technol..

[67] Percy A.E., O’Brien I.E., Jameson P.E., Melton L.D., MacRae E.A., Redgwell R.J. (1996). Xyloglucan endotransglycosylase activity during fruit development and ripening of apple and kiwifruit. Physiol. Plant..

[68] Han Y., Ban Q., Li H., Hou Y., Jin M., Han S., Rao J. (2016). DkXTH8, a novel xyloglucan endotransglucosylase/hydrolase in persimmon, alters cell wall structure and promotes leaf senescence and fruit postharvest softening. Sci. Rep..

[69] Witasari L.D., Huang F.C., Hoffmann T., Rozhon W., Fry S.C., Schwab W. (2019). Higher expression of the strawberry xyloglucan endotransglucosylase/hydrolase genes Fv XTH 9 and Fv XTH 6 accelerates fruit ripening. Plant J..

[70] Wang D., Lu Q., Wang X., Ling H., Huang N. (2023). Elucidating the role of SlXTH5 in tomato fruit softening. Hortic. Plant J..

[71] Ma M., Yuan Y., Cheng C., Zhang Y., Yang S. (2021). The MdXTHB gene is involved in fruit softening in ‘Golden Del. Reinders’ (*Malus pumila*). J. Sci. Food Agric..

[72] Zhang Z., Wang N., Jiang S., Xu H., Wang Y., Wang C., Li M., Liu J., Qu C., Liu W. (2017). Analysis of the xyloglucan endotransglucosylase/hydrolase gene family during apple fruit ripening and softening. J. Agric. Food Chem..

[73] Wang J.H., Sun Q., Ma C.N., Wei M.M., Wang C.K., Zhao Y.W., Wang W.Y., Hu D.G. (2024). MdWRKY31-MdNAC7 regulatory network: Orchestrating fruit softening by modulating cell wall-modifying enzyme MdXTH2 in response to ethylene signalling. Plant Biotechnol. J..

[74] Yang L., Cong P., He J., Bu H., Qin S., Lyu D. (2022). Differential pulp cell wall structures lead to diverse fruit textures in apple (*Malus domestica*). Protoplasma.

[75] Liu H., Qian M., Song C., Li J., Zhao C., Li G., Wang A., Han M. (2018). Down-regulation of PpBGAL10 and PpBGAL16 delays fruit softening in peach by reducing polygalacturonase and pectin methylesterase activity. Front. Plant Sci..

[76] Ban Q., Han Y., He Y., Jin M., Han S., Suo J., Rao J. (2018). Functional characterization of persimmon β-galactosidase gene DkGAL1 in tomato reveals cell wall modification related to fruit ripening and radicle elongation. Plant Sci..

[77] Smith D.L., Abbott J.A., Gross K.C. (2002). Down-regulation of tomato β-galactosidase 4 results in decreased fruit softening. Plant Physiol..

[78] Paniagua C., Blanco-Portales R., Barceló-Muñoz M., García-Gago J.A., Waldron K.W., Quesada M.A., Muñoz-Blanco J., Mercado J.A. (2016). Antisense down-regulation of the strawberry β-galactosidase gene FaβGal4 increases cell wall galactose levels and reduces fruit softening. J. Exp. Bot..

[79] Wang M., Zhan W., Chen M., Guo Y., Wang H., Wu Y., Bai T., Jiao J., Song C., Shi J. (2024). MdAP2-like, a new regulator in apple, simultaneously modulates fruit softening and size. Postharvest Biol. Technol..

[80] Nobile P.M., Wattebled F., Quecini V., Girardi C.L., Lormeau M., Laurens F. (2011). Identification of a novel α-L-arabinofuranosidase gene associated with mealiness in apple. J. Exp. Bot..

[81] Yang H., Liu X., Hu W., Yang Z., Zhao Z. (2025). Apple transcription factor MdDof43 promotes fruit softening by regulating cell wall-modifying genes Mdβ-Gal2 and Mdα-AF3. Hortic. Plant J..

[82] Wang W., Yu J., Du M., Wang J., Hu D. (2022). Basic helix-loop-helix (bHLH) transcription factor MdbHLH3 negatively affects the storage performance of postharvest apple fruit. Hortic. Plant J..

[83] Zhou Q., Zhang F., Ji S., Dai H., Zhou X., Wei B., Cheng S., Wang A. (2021). Abscisic acid accelerates postharvest blueberry fruit softening by promoting cell wall metabolism. Sci. Hortic..

[84] Wu M., Liu K., Li H., Li Y., Zhu Y., Su D., Zhang Y., Deng H., Wang Y., Liu M. (2023). Gibberellins involved in fruit ripening and softening by mediating multiple hormonal signals in tomato. Hortic. Res..

[85] Zhao Y.-W., Zhao T.-T., Sun Q., Liu X.-L., Huang X.-Y., Li L.-G., Wang H.-B., Li W.-K., Wang C.-K., Wang W.-Y. (2025). Enrichment of two important metabolites D-galacturonic acid and D-glucuronic acid inhibits MdHb1-mediated fruit softening in apple. Nat. Plants.

[86] Kou X., Feng Y., Yuan S., Zhao X., Wu C., Wang C., Xue Z. (2021). Different regulatory mechanisms of plant hormones in the ripening of climacteric and non-climacteric fruits: A review. Plant Mol. Biol..

[87] Saladié M., Matas A.J., Isaacson T., Jenks M.A., Goodwin S.M., Niklas K.J., Xiaolin R., Labavitch J.M., Shackel K.A., Fernie A.R. (2007). A reevaluation of the key factors that influence tomato fruit softening and integrity. Plant Physiol..

[88] Veraverbeke E.A., Verboven P., Van Oostveldt P., Nicolaı B.M. (2003). Prediction of moisture loss across the cuticle of apple (*Malus sylvestris* subsp. Mitis (Wallr.)) during storage: Part 1. Model development and determination of diffusion coefficients. Postharvest Biol. Technol..

[89] Bai J., Baldwin E.A., Hagenmaier R.H. (2002). Alternatives to shellac coatings provide comparable gloss, internal gas modification, and quality for ‘Delicious’ apple fruit. HortScience.

[90] Shackel K.A., Greve C., Labavitch J.M., Ahmadi H. (1991). Cell turgor changes associated with ripening in tomato pericarp tissue. Plant Physiol..

[91] Fernández V., Khayet M., Montero-Prado P., Heredia-Guerrero J.A., Liakopoulos G., Karabourniotis G., Del Rio V., Domínguez E., Tacchini I., Nerín C. (2011). New insights into the properties of pubescent surfaces: Peach fruit as a model. Plant Physiol..

[92] Mukhtar A., Damerow L., Blanke M. (2014). Non-invasive assessment of glossiness and polishing of the wax bloom of European plum. Postharvest Biol. Technol..

[93] Wang J., Hao H., Liu R., Ma Q., Xu J., Chen F., Cheng Y., Deng X. (2014). Comparative analysis of surface wax in mature fruits between Satsuma mandarin (*Citrus unshiu*) and ‘Newhall’ navel orange (*Citrus sinensis*) from the perspective of crystal morphology, chemical composition and key gene expression. Food Chem..

[94] Gao H., Yang S., Chen H., Chu W., Mu H., Ge L. (2014). Epicuticular wax’s effect on fruit softening of blueberry. J. Chin. Inst. Food Sci. Technol..

[95] Özgen M., Palta J.P., Smith J.D. (2002). Ripeness stage at harvest influences postharvest life of cranberry fruit: Physiological and anatomical explanations. Postharvest Biol. Technol..

[96] Dong X., Rao J., Huber D.J., Chang X., Xin F. (2012). Wax composition of ‘Red Fuji’ apple fruit during development and during storage after 1-methylcyclopropene treatment. Hortic. Environ. Biotechnol..

[97] Dong X., Rao J., Zhu S., Yang Q. (2013). Combination of modified atmosphere packaging and 1-methylcyclopropene treatment suppress decreasing of wax composition of apples during cold storage. Trans. Chin. Soc. Agric. Eng..

[98] Cajuste J.F., González-Candelas L., Veyrat A., García-Breijo F.J., Reig-Armiñana J., Lafuente M.T. (2010). Epicuticular wax content and morphology as related to ethylene and storage performance of ‘Navelate’ orange fruit. Postharvest Biol. Technol..

[99] Curry E. (2008). Effects of 1-MCP applied postharvest on epicuticular wax of apples (*Malus domestica* Borkh.) during storage. J. Sci. Food Agric..

[100] Knoche M., Lang A. (2017). Ongoing growth challenges fruit skin integrity. Crit. Rev. Plant Sci..

[101] Zhang L., Zhu M., Ren L., Li A., Chen G., Hu Z. (2018). The SlFSR gene controls fruit shelf-life in tomato. J. Exp. Bot..

[102] Jiang F., Lopez A., Jeon S., de Freitas S.T., Yu Q., Wu Z., Labavitch J.M., Tian S., Powell A.L., Mitcham E. (2019). Disassembly of the fruit cell wall by the ripening-associated polygalacturonase and expansin influences tomato cracking. Hortic. Res..

[103] Moctezuma E., Smith D.L., Gross K.C. (2003). Antisense suppression of a β-galactosidase gene (TB G6) in tomato increases fruit cracking. J. Exp. Bot..

[104] Yu J., Wang R., Ma W., Lei S., Zhu M., Yang G. (2023). Pectate lyase gene VvPL1 plays a role in fruit cracking of table grapes. J. Agric. Food Chem..

[105] Maguire K.M., Lang A., Banks N.H., Hall A., Hopcroft D., Bennett R. (1999). Relationship between water vapour permeance of apples and micro-cracking of the cuticle. Postharvest Biol. Technol..

[106] Zheng X., Yuan Y., Huang B., Hu X., Tang Y., Xu X., Wu M., Gong Z., Luo Y., Gong M. (2022). Control of fruit softening and ascorbic acid accumulation by manipulation of SlIMP3 in tomato. Plant Biotechnol. J..

[107] Catiempo R.L., Photchanachai S., Powell A.F., Strickler S.R., Wongs-Aree C. (2024). Transcriptome analysis suggests the role of expansin genes in the improved germination of sunflower (*Helianthus annuus* L.) seeds after hydropriming. Crop Sci..

[108] Cao X., Li M., Li J., Song Y., Zhang X., Yang D., Li M., Wei J. (2021). Co-expression of hydrolase genes improves seed germination of Sinopodophyllum hexandrum. Ind. Crops Prod..

[109] Peng Z., Liu G., Li H., Wang Y., Gao H., Jemrić T., Fu D. (2022). Molecular and genetic events determining the softening of fleshy fruits: A comprehensive review. Int. J. Mol. Sci..

[110] Chen D.-M., Jia L.-G., Zhao G.-D., Zhang C.-H., Yang F.-Q., Zhang X.-S., Zhao T.-S., Li C.-M., Zhao Y.-B. (2022). ‘Yuguan’, a Late-Ripening Apple Cultivar in China. HortScience.

