# Supplementary material for: Advances in Cell Wall Dynamics and Gene Expression in Postharvest Fruit Softening
Source: Plants (Basel). 2025 Sep 10;14(18):2831. doi: 10.3390/plants14182831 (PMC12473168; doi:10.3390/plants14182831)
Supplement: Supplementary file 1 [file plants-14-02831-s001.zip › plants-3800925-supplementary.pdf]

Supplementary Table S1. Modulators Involved in the CWRPs-Mediated Regulatory Network of Fruit Softening

Modulator	Function	Reference
MdPG1	Promotes pectin hydrolysis, water loss and fruit cracking<br>Accelerates fruit softening	[23, 46, 100]
MdEIL2, MdCBF2	Activate the *MdPG1* expression	[43]
MADS6, MADS8, MADS9	Activate the *MdPG1* expression	[47]
MdNAC72	Suppresses the *MdPG1* expression	[3]
MdMAPK3	Phosphorylates MdNAC72 to suppress its transcriptional repression activity	[3]
MdPUB24	Accelerates MdNAC72 degradation by regulating its ubiquitination	[3]
MdPL5	Accelerates fruit softening	[49]
MdZFP3, MdTPL4, MdHDA19	Suppress the expression of cell wall degradation genes, such as *MdPG1* and *MdPL5*	[5]
MdEAEL1	Accelerates MdZFP3 degradation by regulating its ubiquitination	[5]
MdPME2, MdACO4, MdPGLR3	Suppress flesh firmness and/or crispness retainability	[48]
MdERF3	Suppresses the expression of *MdPME2, MdACO4* and *MdPGLR3*	[48]
MdERF118	Suppresses the expression of *MdACO4* and *MdPGLR3*	[48]
MdDOF5.3	Activates the *MdERF3* expression	[48]
MdRAVL1	Activates the *MdERF118* expression	[48]
MdNAC1-L	Activates the expression of *MdPL5*, *MdPG1*, *MdACS1*, and *MdACO1*	[49]
FaPLC, FaPG1, FaPG2	Accelerate fruit softening	[51, 52, 53]
VvPL11	Accelerates fruit softening	[54]
PpPG PpPG21, PpPG22	Accelerate fruit softening	[55, 57]
SlPL,	Accelerates fruit softening, water loss and increases pathogen susceptibility	[56]
SlPG2a	Accelerates fruit softening and cracking	[58, 60, 102]
PpERF/ABR1	Activates the *PpPG* expression	[57]

SlERF.F12, SlTPL2, SlHDA1/3	Suppress the expression of *SlPG2a* and *SlPL*	[58]
SlEXP1	Accelerates fruit softening and potentially increases the substrate accessibility for other cell wall-modifying enzymes	[60]
SlLOB1	Activates the expression of *SlEXP1 and SlPL*	[65]
MdEXLB1	Accelerates reproductive development and fruit softening	[62]
MaC2H2-1, MaC2H2-2	Activate the expression of cell wall-modifying genes, such as *MaEXP-A2, MaEXP-A8, and MaSUR14*	[64]
MdBBX25	Suppresses the expression of cell wall-modifying genes, such as *MdPG, MdCEL, and MdEXPA8*	[66]
DkXTH8	Activates premature leaf aging and fruit softening	[68]
FvXTH9, FvXTH6	Accelerate fruit ripening and softening	[69]
SlXTH5	Accelerates fruit softening	[70]
MdXTHB	Accelerates fruit softening and ethylene production	[71]
MdWRKY31,	Suppresses the *MdXTH2* expression	[73]
MdNAC7	Hinders MdWRKY31 from binding to the promoter of *MdXTH2*	[73]
PpBGAL10, PpBGAL16	Accelerate fruit ripening and softening	[75]
DkGAL1	Accelerates pigmentation shift and fruit softening	[76]
FaβGal4	Decreases cell wall galactose content and accelerates fruit softening	[78]
MdAP2-like, MdZF-HD11	Activate the *Mdβ-GAL18* expression	[7, 79]
MdDof43	Activates the expression of *Mdβ-Gal2 and Mdα-AF3*	[81]
MdbHLH3	enhances the αAFase, β-Gal, and PG activities	[82]
MdMYB2, MdNAC14, MdNTL9	Activate the *MdHb1* expression	[85]
SlFSR	Reduces shelf-life and increases water loss	[101]
SlIMP3	Promotes wall thickening<br>Decreases fruit softening and water loss	[106]

Supplementary Table S2. List of Abbreviations

Abbreviation	Full Term
ACO	1-aminocyclopropane-1-carboxylate oxidase
ACS	1-aminocyclopropane-1-carboxylate synthase
AFase	α-arabinofuranosidase
AP	APETALA
BBX	B-BOX DOMAIN PROTEIN
BGAL	β-galactosidase
bHLH	basic helix-loop-helix
CBF	COLD BINDING FACTOR
CEL	CELLULASE
CWRP	cell wall remodelling protein
Dk	*Diospyros kaki*
DOF	DNA BINDING WITH ONE FINGER
EAEL	ethylene-activated E3 ubiquitin Like
EGase	endo-1,4-β-glucanase
EIL	ETHYLENE-INSENSITIVE3-LIKE
ERF	ETHYLENE RESPONSE FACTOR
EXP	expansin
Fa	*Fragaria × ananassa*
FSR	fruit shelf-life regulator
HDA	HISTONE DEACETYLASE
HGA	homogalacturonan
LOB	LATERAL ORGAN BOUNDARIES
Ma	*Musa acuminata*
MAPK	MITOGEN-ACTIVATED PROTEIN KINASE
MCP	1-Methylcyclopropene
Md	*Malus domestica*
ML	middle lamella
NAC	NAC domain containing protein
PCW	primary cell wall
PG	endo-polygalacturonase
PL	pectate lyase
PME	pectin methylesterase
Pp	*Prunus persica*
PUB	PLANT U-BOX
RAV	RELATED TO ABI3/VP1
RG-I	Rhamnogalacturonan-I
RGL	rhamnogalacturonan lyase
Sl	*Solanum lycopersicum*
TCJ	tricellular junctions
TPL	TOPLESS
VIGS	virus-Induced Gene Silencing
Vv	*Vitis vinifera*

XTH	xyloglucan endo-transglycosylase/hydrolase
ZF-HD	ZINC FINGER HOMEODOMAIN
ZFP	ZINC FINGER PROTEIN
